# Automated separation for heterogeneous immunoassays

**DOI:** 10.1155/S1463924691000111

**Published:** 1991

**Authors:** A. Truchaud, J. Barclay, J. P. Yvert, B. Capolaghi

**Affiliations:** 1 Hôpital de Meaux, BP218 Meaux 77108 France; 2 Bayer International St Denis 93200 France; 3 Hôpital Begin St Mande 94160 France; 4 Hôpital Bel Air Thionville 57100 France

## Abstract

Beside general requirements for modern automated systems, immunoassay automation involves specific requirements as a separation step for heterogeneous immunoassays. Systems are designed according to the solid phase selected: dedicated or open robots for coated tubes and wells, systems nearly similar to chemistry analysers in the case of magnetic particles, and a completely original design for those using porous and film materials.


Journal of Automatic Chemistry, Vol. 13, No. 2 (March–April 1991), pp. 49–51

### Automated separation for heterogeneous immunoassays

**A. Truchaud**

*Hôpital de Meaux, BP218, 77108 Meaux, France*

**J. Barclay**
*Bayer International, 93200 St Denis, France*

**J. P. Yvert**

*Hôpital Begin, 94160 St Mande, France*

**and B. Capolaghi**

*Hôpital Bel Air, 57100 Thionville, France*

*Beside general requirements for modern automated systems, immunoassay automation involves specific requirements as a separation step for heterogeneous immunoassays. Systems are designed according to the solid phase selected: dedicated or open robots for coated tubes and wells, systems nearly similar to chemistry analysers in the case of magnetic particles, and a completely original design for those using porous and film materials.*

### Introduction

The field of immunoassay has shown enormous growth in the last two decades and this has led to a need to automate. The principal advantages of using automated systems include:

(1) Avoidance of tedious and repetitive manual procedures.
(2) Control of reaction conditions significantly improving performance.
(3) Limitation of biohazards.

The automation of the immunological reaction is discussed under three headings: Heterogeneous versus homogeneous immunoassays; Constraints in automation of the immunological reaction; and Automated separation using different solid phases. The authors then identify some new trends in this area.

### Heterogeneous versus homogeneous immunoassays

In homogeneous immunoassays, antibody binding leads to a change in the signal, which is measurable from a label, or from matter in competition for binding with the substance being measured.

The classical example is EMIT: the enzyme immunoassay [1], where the specimen antigen competes with a labelled conjugate. CEDIA, cloned enzyme donor immunoassays, is another example [2].

Immunoprecipitation and immunoagglutination are also used for the quantitation of small molecules, both directly and as inhibition processed, with turbidimetric or nephelometric detection.

In heterogeneous immunoassays, antibody-bound and free analyte must be separated. The antibody is usually fixed on a solid phase and other components are usually added sequentially. Antigens with several binding sites allow assays in 'sandwich' form, i.e. a sandwich with the antigen and a conjugate with an antibody which combines with another epitope of the antigen and the label: this is an extraction process. This technique also allows competition between a free antigen and an antigen-labelled antibody conjugate. In these two cases, the components which are not bound are eliminated by a separation step, which usually also involves washing steps.

The main features of the two kinds of immunological reactions can be summarized as follows:

(1) The reaction mode: competition for homogeneous immunoassays, competition and extraction for heterogeneous immunoassays.
(2) A separation step for heterogeneous immunoassays.
(3) In the homogeneous mode, the modulation of the signal is measured; in the heterogeneous mode, the total remaining signal is measured.
(4) With homogeneous immunoassays, it is possible to determine high concentration analytes with low molecular weight.
(5) Heterogeneous immunoassays can show similar sensitivity and imprecision as radioimmunoassays, with no restriction on molecular size.

### Constraints in the automation of the immunological reaction

In the homogeneous mode, there are only two analytical steps (reagent addition, incubation and measurement), which is similar to most chemical assays. Many of these methods can be adapted to conventional chemistry analysers.

In the heterogeneous mode, there are many analytical steps (see table 1). The separation step is a specific requirement, not found in conventional chemistry analysers. The constraints to automation are:

(1) Choosing, designing and manufacturing a solid phase which is equivalent to a reagent.
(2) Reducing the duration of the incubation steps because if $t_1$ and $t_2$ are 1 h, a very large incubator with more than 300 places will be necessary.
(3) Combining and automating the different components in a compact automatic system. Such systems are often based on patented components or analytical procedures.

0142–0453/91 $3.00 © 1991 Taylor & Francis Ltd.

*Table 1. Constraints in the automation of heterogeneous immunoassays.*

| Analytical steps | Constraints |
|---|---|
| Specimen + reagents | |
| ↓ | * Choice of a solid phase |
| Incubation $t_1$ | |
| ↓ | |
| Separation | |
| ↓ | * $t_1 + t_2 \rightarrow$ Capacity of the incubator |
| + Reagent | |
| ↓ | |
| Incubation $t_2$ | |
| ↓ | |
| Measurement | * Separation |

### Automated separation using different solid phases

Only a few analytical systems are well established: there are several currently being evaluated and developed.

The solid phase is shown in figure 1 with the antibody, the bound and the solid support. The main solid phases are: tubes, wells or beads, latex and magnetic particles, fibrous materials, and, more recently, films.

*Coated tubes, wells or beads*

The advantages of these classical solid phases for manufacturers are that immunological reactions and manufacturing processes are similar to those for radioimmunoassays; furthermore, the shape and size of the microtitre plate is a 'standard', for which robotic processing is readily available. The drawbacks are a large physical volume because each analyte needs a specific coated tube or well, complex mechanical process control and often long incubation delays. Automation is possible through dedicated or reprogrammable robots.

A recent improvement in tube technology is to use 'universal' tubes coated with strepavidin and a biotinilated antibody specific to the antigen, then an enzyme conjugate. This is called the 'soluble sandwich' approach [3].

To accelerate the reaction by increasing the rate of immunological events, the size and geometric space in which the components diffuse can be reduced, the antibody concentration increased and the solid phase split into microparticles.

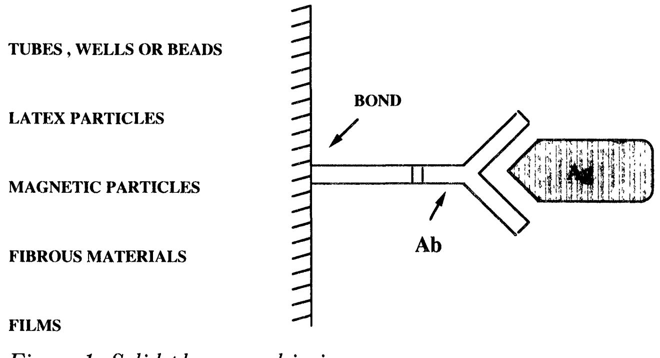

*Figure 1. Solid phases used in immunoassays.*

*Latex particles*

In the Abbott IMX, the latex microparticles of 0·5 μ size react with the specimen antigen, then with the enzyme conjugate; the reaction mixture is transferred in a glass fibre filter where latex particles are captured and then washed [4]. On addition of a fluorescence generating substrate, it is possible to quantitate the immunocomplexes.

*Magnetic particles*

Magnetic particles seem very attractive, because, at first sight, the separation process is simple. In fact, magnetic particles are 'high tech' products with very precise requirements:

(1) Limited non-specifi[illegible] binding.
(2) Stability of the suspension of these particles.
(3) Avoidance of capturing residual liquid during the separation process ('entrapment').
(4) Delay for resuspension.
(5) Avoidance of loss of particles during washing.

It is possible to meter and deliver the suspension in optical cuvettes of a system very similar to a chemistry analyser, except that magnets are located near the cuvettes [5]. The cuvettes are moved in front of the magnets and the separation and washing steps are performed when particles are held in place by the magnets.

It is also possible to measure an absorbance when the magnetic particles are fixed.

*Fibrous and film materials*

It is possible to fix antibodies on fibrous and film materials. The immunological events are induced by moving the other components by diffusion through the solid in a standardized physical process.

A first approach is to use radial diffusion through a porous material (for example the Baxter Stratus system). The unbound reagents are eliminated on the periphery, the bound immunological complexes stay in the centre [6]. The automation is performed with a conveyor belt moving the slides under different dispensers and the fluorimeter, in a temperature-controlled area.

In the Opus system, developed by PB Diagnostics (a venture between Polaroïd and Behring Diagnostics), two different devices are used: one for a film technology, the other for Elisa processes [7]. In the Elisa device, antibodies are fixed in a glass-fibre support and the specimen is delivered onto it. Then the conjugate and washing solutions are delivered and diffused through the glass-fibre matrix. In the other device, a film technology is used. The basic principle is a competition between the antigen and a fluorescent conjugate towards antibodies fixed in a 'signal layer'.

The film is composed of different layers:

(1) The undiluted sample is applied in the spreader, then it hydrates the layer.

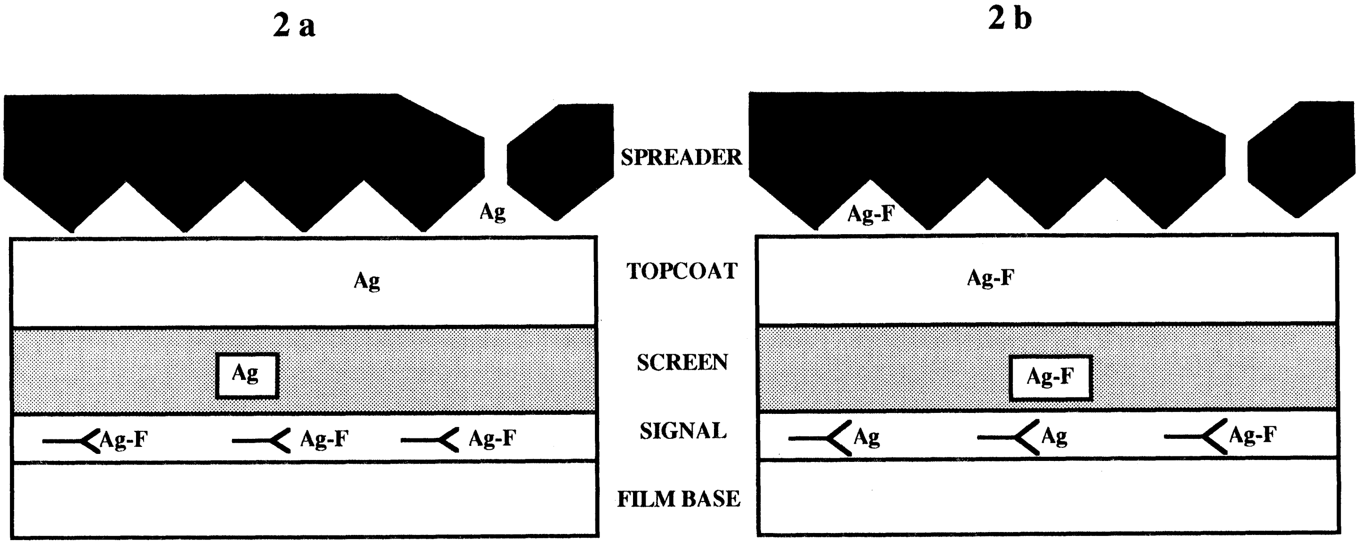

*Figure 2. Multilayer structure and test procedure.*
*Multilayer structure: Ag-F = complex between monoclonal antibody and hapten-fluorescer conjugate. Test procedure: (2*a*) sample rehydrates layers, antigen (Ag) diffuses towards signal layer; (2*b*) antigen displaces conjugate, free conjugate diffuses out of signal layer. Reprinted from Grenner* et al., *Clinical Chemistry (1989).*

(2) Big molecules are displaced and retained in the topcoat layer.
(3) Sample antigen travels through the screen layer and into the signal layer where it displaces labelled antigen as seen before.
(4) The unbound antigen-fluorescent conjugate diffuses freely and is distributed evenly in the layers. The red pigment in the screen layer blocks excitation of the label outside the signal layer. Fluorescence is measured through the polyester fibre base.

Given the physical constraints of the solid phase supports, the incubation times and optical detection, this approach is more adequate for short series. It does not allow continuous loading of specimens and is unsuitable for large volumes.

### Discussion

With automated separation, heterogeneous immunoassays can easily be performed in large batches.

Targets for improved automation include:
(1) Improved reagent stability in the system to about one or two months.
(2) Multiparametric calibration solutions.
(3) Faster reactions which allow compact automation.
(4) Combination of homogeneous and heterogeneous immunoassays in one system.
(5) Development of new solid phases like biosensors.

### References

1. Rubenstein, K. E., Schneider, R. S. and Ullman, U. F., *Biochemical and Biophysical Research Communications*, **47** (1972), 846.
2. Henderson, R., Friedman, B., Harris, D., Manning, B. and Zoccoli, A., *Clinical Chemistry*, **32** (1986), 1637.
3. Chieregatti, G., Massaglia, A., Giraudi, G., Zannino, M., Barbieri, U. and Boniolo, A., *Annales de Biologie Clinique*, **48** (1990), 393.
4. Brown, III, W.E., *Clinical Chemistry*, **33** (1987), 1567.
5. Boland, J., Carey, G., Krodel, E. and Kwiatkowski, M., *Clinical Chemistry*, **36** (1990), 1598.
6. Giegel, J. L., Brotherton, M. M., Cronin, P., D'Aquino, M., Evans, S., Heller, Z. H., Knight, W. S., Krishnan, K. and Sheiman, M., *Clinical Chemistry*, **28** (1982), 1894.
7. Grenner, G., Inbar, S, Meneghini, F. A., Long, E. W., Yamartino, E. J., Bowen, M. S., Blackwood, J. J., Padila, A. J., Maretsky, D. and Staedter, M., *Clinical Chemistry*, **35** (1989), 1865.

